# Hepatocyte-specific knockout of HIF-2α cannot alleviate carbon tetrachloride-induced liver fibrosis in mice

**DOI:** 10.7717/peerj.15191

**Published:** 2023-04-03

**Authors:** Jianfang Ye, Jie Chen, Yun Li, Liao Sun, Hongyun Lu

**Affiliations:** 1 Department of Endocrinology and Metabolism, The Fifth Affiliated Hospital of Sun Yat-sen University, Zhuhai, Guangdong, China; 2 Molecular Imaging Center, The Fifth Affiliated Hospital of Sun Yat-sen University, Zhuhai, Guangdong, China; 3 Department of Gastroenterology, The Fifth Affiliated Hospital of Sun Yat-sen University, Zhuhai, Guangdong, China; 4 Department of Endocrinology and Metabolism, Zhuhai Hospital Affiliated with Jinan University, Zhuhai, Gangdong, China

**Keywords:** HIF-2α, Liver fibrosis, Liver injury, Lipid metabolism

## Abstract

**Background:**

The effects of hypoxia inducible factor-2α (HIF-2α) deficiency on liver fibrosis have not been demonstrated in a fibrosis model induced by carbon tetrachloride (CCl_4_). We aimed to examine whether hepatocyte-specific HIF-2α deletion could ameliorate CCl_4_-induced liver fibrosis in mice.

**Methods:**

Hepatocyte-specific HIF-2α knockout mice were created using an albumin promoter-driven Cre recombinase. HIF-2α knockout (KO) mice and floxed control wild-type (WT) mice were fed a normal diet (ND) and received either twice weekly intraperitoneal injections of CCl_4_ solution (CCl_4_ dissolved in olive oil) or the corresponding amount of olive oil for 8 weeks. The indicators of liver function, glucose and lipid metabolism, and liver histology were compared among the different groups.

**Results:**

Hepatocyte-specific HIF-2α knockout had no effect on the growth, liver function, glucose or lipid metabolism in mice. CCl_4_-treated KO and WT mice had a similar pattern of injury and inflammatory cell infiltration in the liver. Quantification of Masson staining, α-smooth muscle actin (α-SMA) immunohistochemistry, and the hydroxyproline (HYP) content revealed similar liver fibrosis levels between KO and WT mice injected intraperitoneally with CCl_4_. Immunohistochemistry analysis suggested that HIF-2α was mainly expressed in the portal area and hepatic sinusoids but not in hepatocytes. Bioinformatics analyses further indicated that HIF-2α expression was neither liver specific nor hepatocyte specific, and the effect of HIF-2α in hepatocytes on liver fibrosis may not be as important as that in liver sinuses.

**Conclusions:**

Hepatocyte HIF-2α expression may not be a key factor in the initiation of liver fibrogenesis, and hepatocyte-specific deletion of HIF-2α may not be the ideal therapeutic strategy for liver fibrosis.

## Introduction

Chronic liver diseases (CLD) are characterized by long-term chronic parenchymal injury, persistent activation of the inflammatory response, and sustained activation of liver fibrogenesis and the wound healing response (Parola & Pinzani, 2019). CLD represents a major concern for public health worldwide, with more than 800 million people affected and a mortality rate of approximately two million deaths per year (Asrani et al., 2019). In addition, decompensated cirrhosis accounts for approximately one million deaths per year worldwide and 170,000 deaths per year in Europe. Furthermore, liver cirrhosis accounts for 75–80% of primary liver malignancies and more than 5,000 liver transplantations (Mcglynn, Petrick & El-Serag, 2021; Sung et al., 2021).

Liver fibrosis represents an attempt to limit the consequences of chronic liver injury, although it represents key features of the progression of any form of CLD toward liver cirrhosis. Inhibiting liver fibrosis is a critical therapeutic strategy for treating CLD. Considering that chronic intermittent hypoxia is an important pathophysiological process in CLD (Wu et al., 2019), hypoxia-inducible factors (HIFs), the most important responders of hypoxia, have been shown to be key regulators in liver fibrosis by activating hepatic stellate cells, releasing inflammatory mediators, inducing hepatic sinusoidal capillarization and angiogenesis, mediating chronic inflammation, and epigenetic modification (Wu et al., 2019; Mesarwi et al., 2021; Cai et al., 2021). HIF is composed of one oxygen-dependent α subunit and one constitutively expressed β subunit. When cells are exposed to hypoxia, the hypoxia-sensitive α subunit (HIF-1α, HIF-2α, or HIF-3α) combines with the β subunit (HIF-1β), thus forming a complex capable of activating hypoxia response elements. This mechanism of HIF activates various fibrogenic factors and drives liver fibrosis (Mcgettrick & O’Neill, 2020; Nath & Szabo, 2012; Foglia et al., 2021).

Previous studies have shown that HIF-1α is ubiquitously expressed in all cells, while HIF-2α is more selectively expressed in hepatocytes, endothelial cells, and myocardial cells (Foglia et al., 2021; Patel & Simon, 2008; Schödel & Ratcliffe, 2019). In addition, studies have shown that HIF-1α expression is induced by acute hypoxia and that its response lasts for the first 24 h, whereas HIF-2α is induced by mild hypoxia and lasts for a longer time (Schödel & Ratcliffe, 2019; Semenza, 2012). Therefore, HIF-2α may have a crucial role in mediating the progression of CLD. Several studies have reported that HIF-2α drives liver fibrosis by augmenting lipid accumulation, inflammation, and histidine-rich glycoprotein (Cai, Bai & Ge, 2021; Qu et al., 2011; Morello et al., 2018), but this evidence has not been supported by the fibrosis model induced by carbon tetrachloride (CCl_4_). Therefore, we aimed to explore whether hepatocyte-specific HIF-2α knockout could improve CCl_4_-induced liver fibrosis in mice.

## Materials and Methods

### Animals and treatments

We used the Cre-loxP system to generate hepatocyte-specific HIF-2α knockout mice. In principle, the albumin promoter drives the expression of cyclization recombination enzyme (Cre), leading to HIF-2α knockout (KO) located between two floxed (fl) sequences in hepatocytes. Floxed control wild-type (WT) mice were used as the control group. The mice used in this experiment were on the C57BL/6J background. Epas1tm1Mcs/J mice (HIF-2α^fl/fl^) were purchased from the Jackson Laboratory (Bar Harbor, ME, USA; stock #008407), and B6.Cg-Speer6-ps1Tg 21Mgn/J mice (Alb-Cre^+/−^) were purchased from Shanghai Model Organisms Center, Inc. (Shanghai, China; stock #JAX-003574). All mice were kept in an experimental condition with a 12-h light-to-dark cycle at 25 °C ± 2 °C and were fed a normal diet (ND) and water *ad libitum*. Animal studies were performed according to protocols approved by the Institutional Animal Care and Use Committee of the Fifth Affiliated Hospital of Sun Yat-sen University (approval number: 00158). All mice were treated humanely and with efforts to minimize suffering. Mice were deeply anesthetized with isoflurane and sacrificed by cervical dislocation. There were no surviving animals at the end of study.

Animal experiments were divided into two stages. In the first stage, eleven 7-week-old male mice (six KO and five WT mice) were fed a ND for 16 weeks to examine the possible effect of hepatocyte-specific HIF-2α deletion on the growth and metabolic indexes of mice, the body weight and food intake of mice were measured every twice week. In the second stage, thirty 7-week-old male mice (fifteen KO and fifteen WT mice) were divided into four groups [WT (*n* = 8), KO (*n* = 8), WT + CCl_4_ (*n* = 7), and KO + CCl_4_ (*n* = 7)] to evaluate whether hepatocyte-specific HIF-2α deletion could improve CCl_4_-induced liver fibrosis in mice, the body weight and food intake of mice were measured every week. Groups treated with CCl_4_ were intraperitoneally injected with 10% CCl_4_ (CCl_4_: olive oil = 1:9) at 5 ml/kg for 8 weeks, twice a week, to develop a liver fibrosis model, and the groups treated without CCl_4_ were intraperitoneally injected with the corresponding amount of olive oil.

### General condition and body composition analysis

An EchoMRI quantitative magnetic resonance (QMR) system (Nixon et al., 2010) (EchoMRI-500H; EchoMRI Houston, TX, USA) was used to analyze the body composition (fat mass, lean mass, total body water and free water) of mice in the awake state.

### Glucose and insulin tolerance

We performed intraperitoneal glucose tolerance test (IPGTT) and intraperitoneal insulin tolerance test (IPITT) to evaluate glucose and insulin tolerance 2 weeks before the mice were sacrificed. Mice were injected intraperitoneally with 0.75 U/kg insulin after 4 h of fasting or 2.5 g/kg glucose after 12 h of fasting. Blood glucose from the tail tip was measured at 0, 30, 60, 90, and 120 min after injection using a Roche blood glucose monitoring system. Serum insulin levels were quantitatively measured by a commercial ELISA kit (Mercodia Mouse Insulin ELISA, 10-1247-10; Mercodia, Uppsala, Sweden). Insulin resistance index (HOMA-IR) was calculated using fasting serum glucose and fasting serum insulin according to the previously mentioned article (Yu et al., 2023).

### Blood parameter analyses

After 8 h of fasting, all mice were sacrificed, and blood samples were collected. After centrifugation at 1,000×*g* for 15 min at 4 °C, serum samples were collected and stored at −80 °C until measurements. Serum levels of alanine aminotransferase (ALT), aspartate aminotransferase (AST), total cholesterol (TC), triglyceride (TG), high-density lipoprotein (HDL), and low-density lipoprotein (LDL) were measured according to the manufacturer’s protocols using commercial quantification assay kits (Nanjing Jiancheng Bioengineering Institution, Nanjing, China).

### Liver hydroxyproline measurements

Hydroxyproline levels in the liver tissues were measured using commercial quantification assay kits from Nanjing Jiancheng Bioengineering Institution (Nanjing, China) according to the manufacturer’s protocols.

### Liver histology

Liver tissues were fixed in 4% paraformaldehyde for 24 h, dehydrated and embedded in paraffin. Paraffin sections (4-μm thick) were stained with Masson’s trichrome to evaluate the degree of collagen distribution. Liver fibrosis was scored with blinding of genotype using the fibrosis ISHAK score. The ISHAK staging was determined according to the Ishak et al. (1995) protocol. Hematoxylin and eosin (HE) staining was used to evaluate the degree of necrotizing inflammatory liver injury. Immunohistochemistry (IHC) staining of the liver was used to determine the localization and expression of HIF-2α and α-SMA. The HIF-2α antibody (NB100-122; Novus, St. Louis, MO, USA) was used at a dilution of 1:100, and α-SMA antibody (ab124964; Abcam, Cambridge, UK) was used at a dilution of 1:1,000.

### Polymerase chain reaction (PCR)

An alkaline lysis protocol was used to extract genomic DNA from the mouse toe/tail, and the genotype of the mice was confirmed by PCR analysis. The forward primer for HIF-2α was 5′-GAGAGCAGCTTCTCCTGGAA-3′, and the reverse primer for HIF-2α was 5′-TGTAGGCAAGGAAACCAAGG-3′. Two pairs of primers were used for Cre amplification. The first forward primer for Cre was 5′-ATTTGCCTGCATTACCGGTCG-3′, and the corresponding reverse primer was 5′-CAGCATTGCTGTCACTTGGTC-3′, which amplified a 310-bp band. The second forward primer for Cre was 5′-CAAATGTTGCTTGTCTGGTG-3′, and the corresponding reverse primer was 5′-GTCAGTCGAGTGCACAGTTT-3′, which produced 200-bp products. All PCR products were identified by agarose gel electrophoresis.

### Quantitative real-time PCR (qRT–PCR)

Total RNA was extracted from liver tissues using a Tissue Total RNA Isolation Kit following the manufacturer’s instructions (Vazyme Biotech Co., Ltd., Nanjing, China) and then reverse-transcribed to complementary DNA. Finally, qRT‒PCR was performed by a QuantStudio 7 Flex Real-time PCR System. The forward primer for mouse HIF-2α was 5′-AAGGTGAAGAGCATCATAACCCT-3′, and the corresponding reverse primer was 5′-TCACGCCTTTCATAACACATTCC-3′. The forward primer for mouse β-actin was 5′-CTGGCACCACACCTTCTAC-3′, and the corresponding reverse primer was 5′-TCGTAGATGGGCACAGTGTGG-3′.

### Bioinformatics analyses

RNA sequencing (RNA-seq) data of 31 normal human tissues in the Genotype-Tissue Expression (GTEx) database were downloaded from UCSC (https://xenabrowser.net/datapages/). The RNA-seq matrix and gene-expression array matrix of patients with alcoholic-related liver disease (GSE142530), chronic HBV-related liver disease (GSE84044), chronic HCV-related liver disease (GSE33650), and nonalcoholic fatty liver disease (GSE49541) were downloaded from the Gene Expression Omnibus database (https://www.ncbi.nlm.nih.gov/gds/). The RNA-seq matrix of CCl_4_-induced liver fibrosis in mice was from GSE207855. Gene symbols were transformed according to their platform files, and the expression level of duplicate gene symbols in the same sample was taken from their average value. Gene expression levels are shown as transcripts per kilobase per million mapped reads (TPM) and relative mRNA levels for the RNA-seq data and gene-expression array data, respectively. Detailed information about these datasets is shown in Table S1.

Single-cell RNA-seq of hepatic nonparenchymal cells in normal and CCl_4_-induced liver fibrotic mice was performed using GSE134037. The “Seurat” package was used to preprocess the single-cell RNA sequencing data (Butler et al., 2018). Genes expressed in fewer than five cells in a sample and cells that expressed fewer than 600 genes were excluded. Cells whose total feature RNA was more than 4,500, total unique molecular identifiers (UMIs) were more than 10,000, and mitochondrial gene content was >12% of the total UMIs were considered quality control standards. Data processing followed the standard process and used the default parameters. We retained 20 components for the merged object according to the elbow plot. To cluster these cells, we first applied the FindNeighbors function to construct a K-nearest neighbor graph according to the Euclidean distance in PCA space and then performed the FindClusters function to iterate group cells together with the resolution parameter set at 0.5. Furthermore, we contrasted cells from the subcluster to all other cells of that subcluster using the FindMarkers function. Biologically meaningful cell types were annotated according to the DEGs of each subcluster and canonical cell markers based on the “SingleR” package combined with manual work (Aran et al., 2019). All bioinformatics analyses were performed using R software (Version 4.2.0; R Core Team, 2022).

### Statistical analyses

Data are presented as the mean ± standard deviation (SD). Group comparisons were performed using Student’s t test. Statistical analysis was performed using GraphPad Prism 8 (GraphPad Software Inc., San Diego, CA, USA). A two-sided p value less than 0.05 was considered statistically significant.

## Results

### Establishment of hepatocyte-specific HIF-2α knockout mice

**Figure 1:**
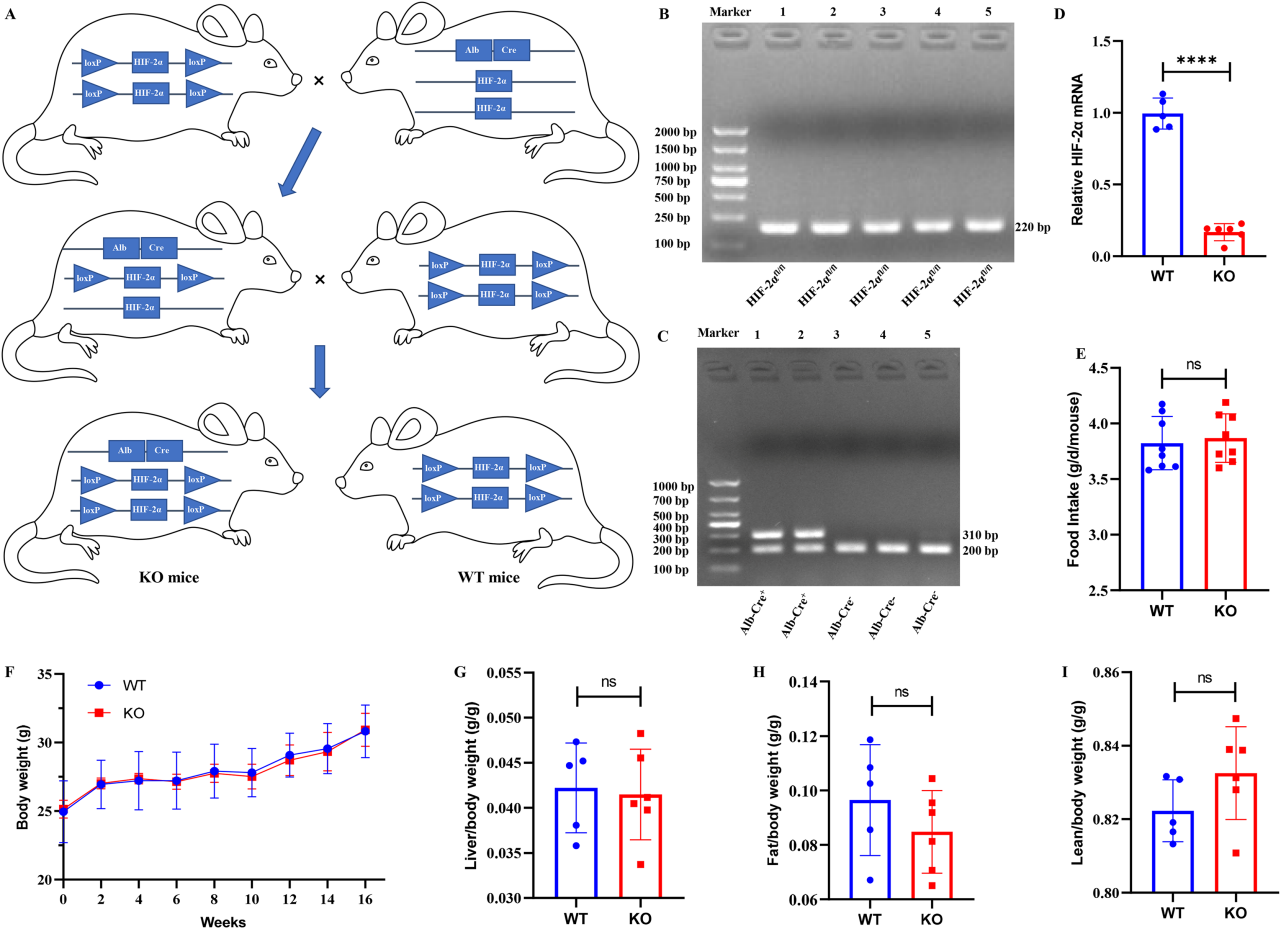
Establishment of hepatocyte-specific HIF-2α knockout mice. (A) Mating process to produce Alb‐Cre^+^/HIF-2α^fl/fl^ (KO) and Alb‐Cre^−^/HIF-2α^fl/fl^ (WT) mice. (B and C) Identification of the genotypes of HIF-2α and the Alb-Cre enzyme in liver tissues by PCR. (D) Relative HIF-2α mRNA in liver tissues detected by qPCR. (E–I) Comparison of food intake (E), body weight (F), liver/body weight (G), fat/body weight (H) and lean/body weight (I) between WT (*n* = 5) and KO mice (*n* = 6). ns, not significant; ****p < 0.0001.

Mice with the HIF-2α^fl/fl^ genotype were mated to Alb-Cre^+^ mice, and we obtained Alb-Cre^+^/HIF-2α^fl/wt^ mice from their offspring. Then, the Alb-Cre^+^/HIF-2α^fl/wt^ mice were mated to Alb-Cre^−^/HIF-2α^fl/fl^ mice to produce Alb-Cre^+^/HIF-2α^fl/fl^ (KO) mice and Alb-Cre^−^/HIF-2α^fl/fl^ (WT) mice (Fig. 1A). The mouse genotypes were identified by PCR. The HIF-2α^fl/fl^ mice produced a 220-bp band for HIF-2α amplification (Fig. 1B), and the Alb-Cre^+^ mice produced hybrid bands of 310 and 200 bp for Cre amplification (Fig. 1C). The relative HIF-2α mRNA level was obviously downregulated in the KO mice (Fig. 1D), which indicated that Alb-Cre^+/−^ mediated recombination of the HIF-2α^fl/fl^ allele disrupted the HIF-2α gene in the hepatocytes. Eleven 7-week-old male mice (six KO and five WT mice) were fed a ND for 16 weeks to examine the possible effect of hepatocyte-specific HIF-2α knockout on the growth of mice. We found that hepatocyte-specific HIF-2α knockout had no impact on food intake (Fig. 1E), body weight (Fig. 1F), liver/body weight (Fig. 1G), fat/body weight (Fig. 1H) or lean/body weight (Fig. 1I).

### Hepatocyte-specific HIF-2α knockout does not impair metabolic indexes

**Figure 2:**
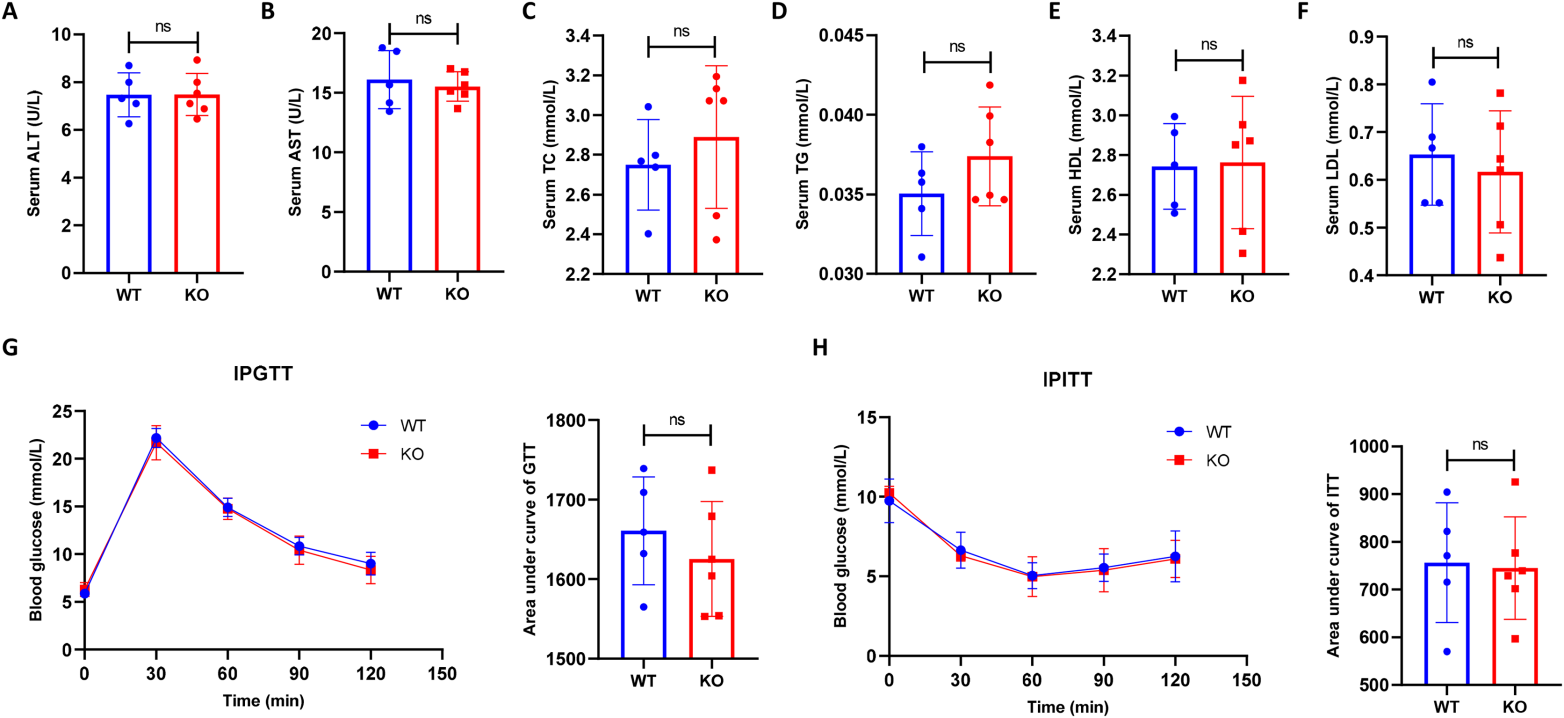
Hepatocyte-specific HIF-2α knockout does not impair the metabolic indexes. (A–F) Comparison of serum alanine aminotransferase (ALT), aspartate aminotransferase (AST), total cholesterol (TC), triglyceride (TG), high-density lipoprotein (HDL), and low-density lipoprotein (LDL) levels between WT (*n* = 5) and KO mice (*n* = 6). (G–J) Comparison of the intraperitoneal glucose tolerance test (IPGTT) and intraperitoneal insulin tolerance test (IPITT) between WT (*n* = 5) and KO mice (*n* = 6). ns, not significant.

To exclude the potential effect of hepatocyte-specific HIF-2α disruption on mouse metabolism, we compared the serum metabolic indexes between the KO and WT mice. As a result, we found that hepatocyte-specific HIF-2α deficiency resulted in no damage to serum ALT, AST, TC, TG, HDL, and LDL levels (Figs. 2A–2F). In addition, the IPGTT and IPITT indicated that hepatocyte-specific HIF-2α deficiency did not affect glucose and insulin tolerance in mice (Figs. 2G and 2H). These initial data showed that hepatocyte-specific HIF-2α knockout had no significant effect on metabolic indexes in mice fed a normal diet.

### Hepatocyte-specific HIF-2α deletion does not alleviate liver injury in liver fibrosis mice

**Figure 3:**
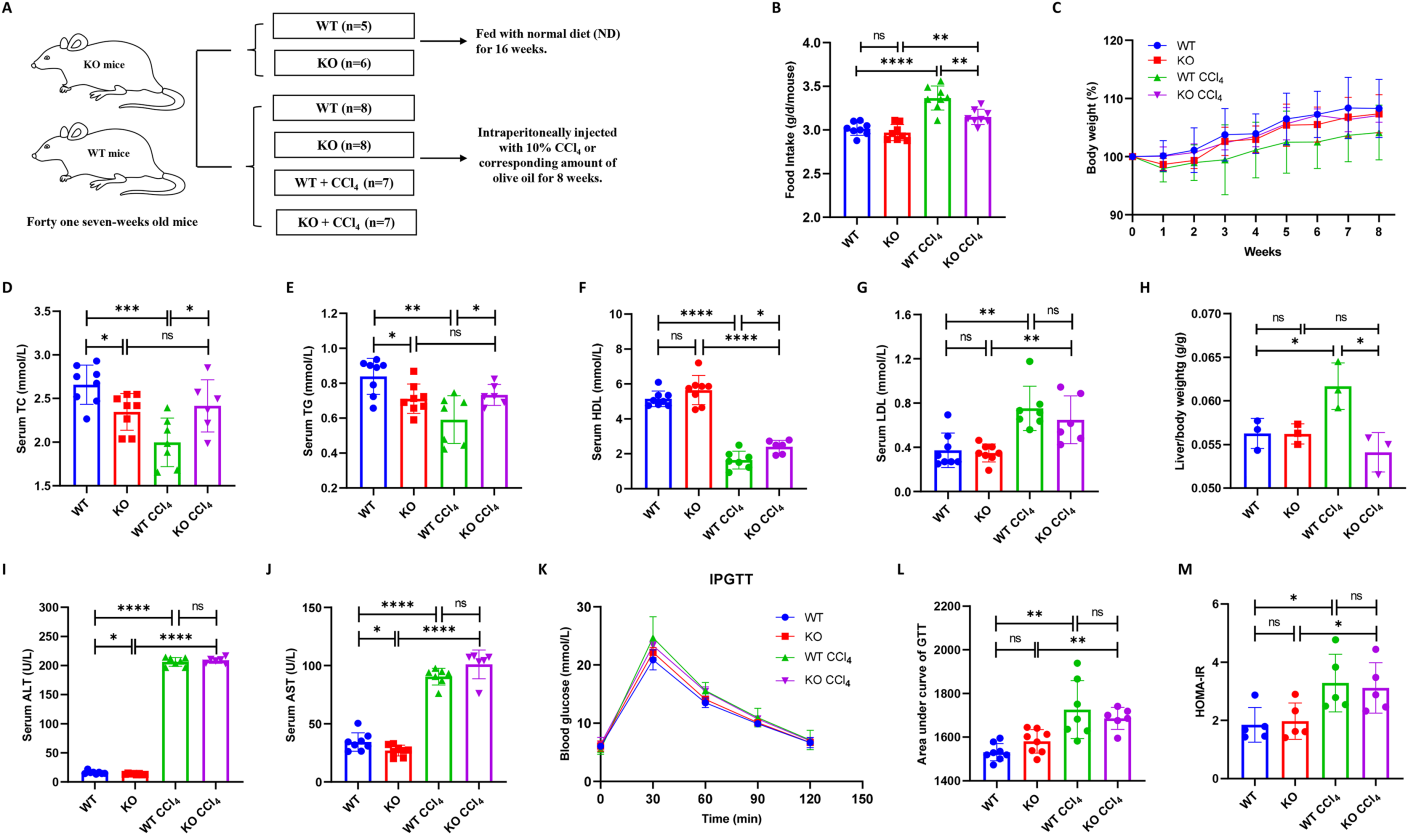
Hepatocyte-specific HIF-2α deletion does not alleviate liver injury in liver fibrosis mice. (A) Grouping and intervention of mice. (B and C) Comparison of the food intake (B) and body weight (C) of mice in different groups. (D–G) Comparison of serum TC (D), TG (E), HDL (F), and LDL (G) levels of mice in different groups. (H) Comparison of liver/body weight of mice in different groups. (I and J) Comparison of serum ALT (I) and AST (J) of mice in different groups. (K–M) Comparison of IPGTT (K), IPITT (L) and HOMA-IR (M) of mice in different groups. ALT, serum alanine aminotransferase; AST, aspartate aminotransferase; TC, total cholesterol; TG, triglyceride; HDL, high-density lipoprotein; LDL, low-density lipoprotein; IPGTT, intraperitoneal glucose tolerance test; IPITT, intraperitoneal insulin tolerance test; HOMA-IR, homeostasis model assessment of insulin resistance. ns, not significant; *p < 0.05; **p < 0.01; ***p < 0.001; ****p < 0.0001.

Thirty male mice (fifteen KO and fifteen WT mice) were divided into four groups: WT (*n* = 8), KO (*n* = 8), WT + CCl_4_ (*n* = 7), and KO + CCl_4_ (*n* = 7). One mouse in the KO + CCl_4_ group died at the second week due to improper intraperitoneal administration. Therefore, twenty-nine mice that completed the entire experiment were finally used for analyses (Fig. 3A). The food intake of mice in the WT + CCl_4_ group was obviously greater than that of mice in the other three groups (Fig. 3B), but their body weight and serum lipid levels were the lowest (Figs. 3C–3E), which suggested that the mice in this group expended more energy fighting against disease. Notably, the serum lipid levels in the KO group were significantly lower than those in the WT group, and the dyslipidemia in the KO + CCl_4_ group was also better than that in the WT + CCl_4_ group (Figs. 3D–3G). Hepatocyte-specific HIF-2α deficiency did not affect serum lipid levels in mice without any treatments (Figs. 1C and 1F), but it improved the serum TC and TG levels in mice injected intraperitoneally with olive oil (Figs. 3D–3E). Conversely, hepatocyte-specific HIF-2α deficiency protected the hepatic lipid synthesis function and caused a higher TC and TG levels in liver fibrosis mice (Figs. 3D–3E). This indicates that hepatocyte-specific HIF-2α knockout may regulate liver lipid synthesis and stabilize serum lipid levels. The liver/body weight in the KO + CCl_4_ group was lower than that in the WT + CCl_4_ group (Fig. 3H), but hepatocyte-specific HIF-2α deletion did not improve liver function (Figs. 3I and 3J) or glucose metabolism disorders (Figs. 3K–3M) in mice with CCl_4_-induced liver fibrosis.

### Hepatocyte-specific HIF-2α deficiency does not improve fibrogenesis in liver fibrosis mice

**Figure 4:**
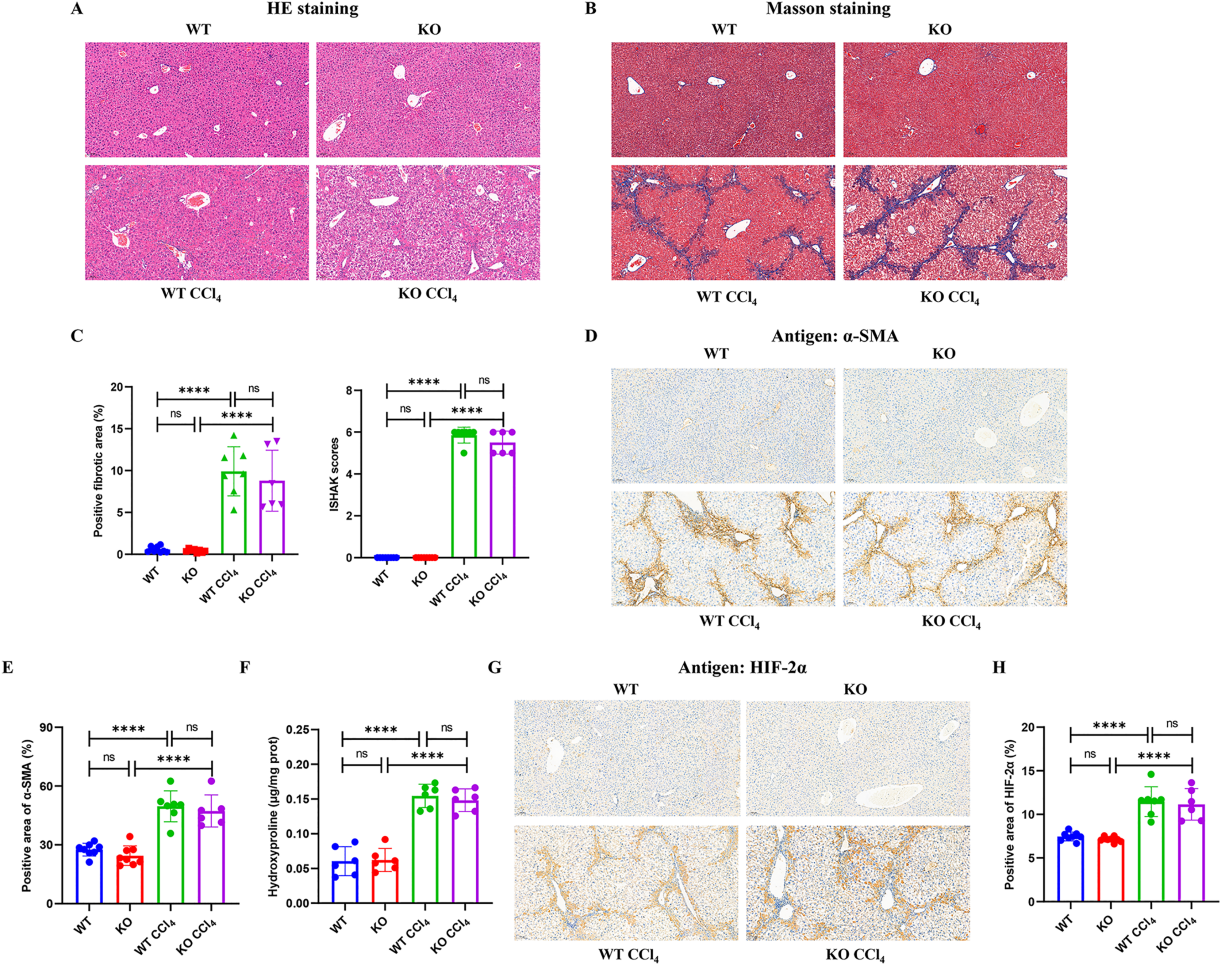
Hepatocyte-specific HIF-2α deficiency does not improve fibrogenesis in liver fibrosis mice. (A) Hematoxylin and eosin (HE) staining of liver tissues (100×, scale bar: 100 μm). (B) Masson staining of liver tissues (100×, scale bar: 100 μm). (C) Positive fibrosis area and ISHAK scores in liver based on Masson staining. (D) Immunohistochemistry staining of α-smooth muscle actin (α-SMA) in liver tissues (100×, scale bar: 100 μm). (E) Percentage of α-SMA-positive area in liver tissues. (F) Hydroxyproline content in liver tissues. (G) Immunohistochemistry staining of HIF-2α in liver tissues (100×, scale bar: 100 μm). (H) Percentage of HIF-2α-positive area in liver tissues. ns, not significant; ****p < 0.0001.

Mice injected intraperitoneally with CCl_4_ developed obvious hepatocyte necrosis, inflammatory cell infiltration, and fiber deposition in the liver according to HE and Masson staining (Figs. 4A and 4B). However, there were no significant differences in the positive fibrotic area (%) and ISHAK scores between the KO + CCl_4_ group and the WT + CCl_4_ group (Fig. 4C). The α-SMA content (Figs. 4D and 4E) and hydroxyproline content (Fig. 4F), which reflected the severity of liver fibrosis, also showed no significant difference between the two groups. These results suggest that hepatocyte-specific HIF-2α knockout could not improve CCl_4_-induced liver fibrosis in mice. Immunohistochemistry staining showed that liver HIF-2α expression increased significantly in mice injected intraperitoneally with CCl_4_, but no difference was observed between the KO + CCl_4_ group and the WT + CCl_4_ group (Figs. 4G and 4H). Notably, we found that increased HIF-2α expression mainly occurred in the portal area and hepatic sinusoids but not in hepatocytes in mice injected intraperitoneally with CCl_4_ (Fig. 4G), which indicates that the hypoxia sites of CCl_4_-induced liver fibrosis are mainly concentrated in the portal area and hepatic sinusoids but not in hepatocytes. This may be the main reason why hepatocyte-specific HIF-2α knockout could not alleviate liver fibrosis. In addition, the increased HIF-2α protein expression was distributed along the hepatic sinuses. Locations close to the portal area demonstrated robust HIF-2α protein expression in the cytoplasm and nucleus of sinus endothelial cells in the fibrotic liver.

### Hepatocyte-specific HIF-2α may not be a key fibrogenic factor in liver fibrosis

**Figure 5:**
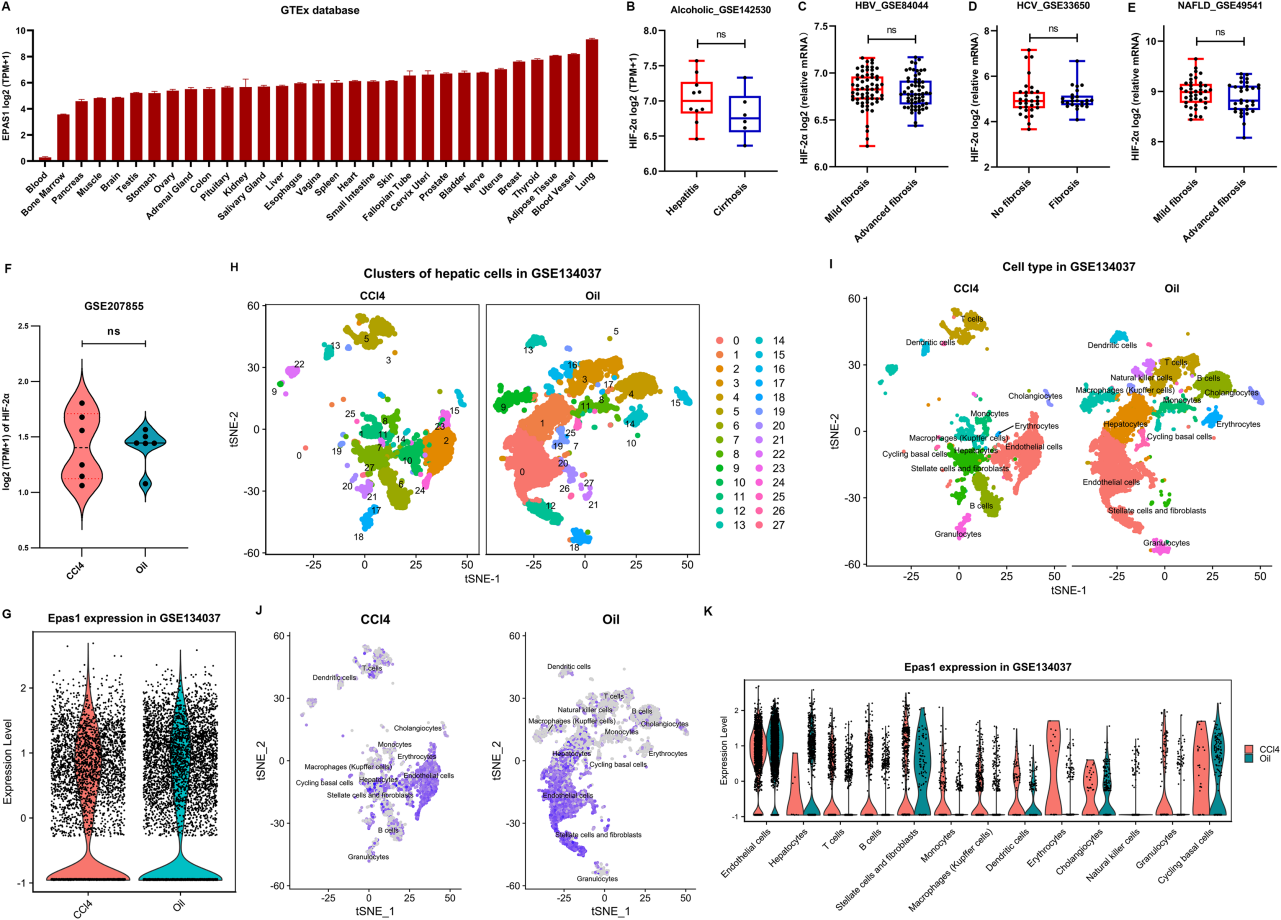
HIF-2α expression in the liver based on bioinformatics analyses. (A) HIF-2α transcription levels in normal tissues in the GTEx database. (B–E) Calculated HIF-2α transcription level in patients with alcoholic-related liver disease (B), chronic HBV-related liver disease (C), chronic HCV-related liver disease (D), and nonalcoholic fatty liver disease (E) based on the GEO database. (F) HIF-2α transcription levels in mice injected intraperitoneally with CCl4 and olive oil. (G) Total HIF-2α transcription levels based on single-cell sequencing data in the GSE134037 dataset. (H and I) Twenty-eight cell clusters were obtained by single-cell sequencing analysis (H), and thirteen cell subsets were identified (I). (J) HIF-2α transcription levels in different cell subsets. The darker the color is, the higher the HIF-2α transcription level. (K) HIF-2α transcription levels in different cell subsets.

HIF-2α has a broad human tissue distribution, with the highest expression in lung and the lowest expression in blood, as suggested in the GETx database (Fig. 5A). However, obviously, it is not a liver-specific protein. In addition, severe liver fibrosis did not result in a significant change in liver HIF-2α transcription levels in patients with alcoholic-related liver disease, chronic HBV-related liver disease, chronic HCV-related liver disease, and nonalcoholic fatty liver disease (Figs. 5B–5E). Similarly, HIF-2α transcription in the liver showed no significant fluctuations in CCl_4_-induced liver fibrosis mice (Fig. 5F), which was different from its protein expression trend, as suggested by our results (Figs. 4G and 4H). This may be because hypoxia affects the protein expression level of HIF-2α rather than its transcription level since pVHL-mediated HIF degradation depends on ambient oxygen.

We further used the single-cell RNA-seq data of liver nonparenchymal cells from CCl_4_-induced fibrosis mice to examine the expression of HIF-2α in different hepatic cells. In terms of total hepatic nonparenchymal cells, there was no difference in HIF-2α transcription between the intraperitoneal injection of CCl_4_ or oil (Fig. 5G). The liver nonparenchymal cells were divided into 28 cell clusters and annotated to 15 cell types (Figs. 5H and 5I). Residual hepatocytes remained in the mice injected intraperitoneally with olive oil, but this did not affect our judgment that the volume of hepatic satellite cells and fibroblasts increased significantly in CCl_4_-induced liver fibrosis mice (Fig. 5I). Notably, HIF-2α mainly existed in sinusoidal endothelial cells but rarely in other cell types (Figs. 5J and 5K). The above results revealed that HIF-2α expression is not liver specific or hepatocyte specific, and knockout of HIF-2α in sinusoidal endothelial cells instead of hepatocytes may improve liver fibrosis.

## Discussion

Liver fibrosis remains an important clinical problem throughout the world. Despite significant advances in understanding the disease, no effective drugs have been developed to directly prevent or reverse the fibrotic process. For many patients, liver transplantation remains the only viable option. Identification of new therapeutic targets that will slow or reverse the progression of fibrosis in such patients is necessary. However, undoubtedly, there is still no efficient target to block this process. Here, we reported that hepatocyte-specific deficiency of HIF-2α cannot improve CCl_4_-induced liver fibrosis in mice, and deletion of hepatocyte-specific HIF-2α is not the ideal therapeutic strategy for liver fibrosis.

The growing interest in HIFs is based on the consensus that in physiological conditions, the liver has a unique double vascular supply in that it receives oxygen-depleted blood from portal vein branches and highly oxygenated blood *via* the hepatic artery. The double vasculature supply generates a gradient of oxygen partial pressure following the direction of the blood flow. Under normal conditions, the liver hypoxic response does not occur, but liver injury induced by alcohol, viruses, drugs, and genetic disorders initiates a hypoxic process and activates HIFs (Copple et al., 2009). In hepatocytes, HIFs promote the release of vascular endothelial growth factor (VEGF) and activate transforming growth factor β (TGF-β). In macrophages, HIFs regulate the production of VEGF, platelet-derived growth factor, and fibroblast growth factor. All the above fibrotic factors promote fibrosis by affecting hepatic stellate cell activation, proliferation, and collagen deposition.

A few studies have shown that HIF-1α deletion in mouse livers has a substantial effect on alleviating liver fibrosis (Mesarwi et al., 2016; Strowitzki et al., 2018; Moon et al., 2009; Copple, Kaska & Wentling, 2012). Notably, acute hypoxia often activates HIF-1α expression, while mild hypoxia promotes activation of HIF-2α expression. Therefore, HIF-2α may be more important to the development of liver fibrosis. Unexpectedly, our study revealed that hepatocyte-specific HIF-2α loss cannot improve CCl_4_-induced liver fibrosis. Similarly, a study reported that hepatocyte-specific deletion of HIF-1β also cannot improve thioacetamide-induced liver fibrosis *in vivo* (Scott et al., 2015), which is similar to our conclusions. The possible reasons why hepatocyte-specific HIF-2α knockout cannot alleviate liver fibrosis may be as follows: (1) HIF-2α is mainly expressed in the lung, and its expression in the liver is not specific; (2) in the liver, HIF-2α is mainly expressed in sinusoidal endothelial cells but not in hepatocytes; (3) liver fibrosis is accompanied by increased HIF-2α expression in nonparenchymal cells instead of hepatocytes; (4) although HIF-2α in hepatocytes was successfully knocked out, HIF-2α was still markedly expressed in the portal region and perivascular area; and (5) although HIF-2α in hepatocytes was successfully knocked out, others HIFs proteins may act independently to driver liver fibrosis.

Qu et al. (2011) reported that HIF-2α promoted steatohepatitis through augmenting lipid accumulation, inflammation and fibrosis, but it did not show the localization of elevated HIF-2α protein in liver. Morello et al. (2018) showed the localization of elevated HIF-2α protein in NAFLD-driven liver. Unfortunately, this study did not show the expression of HIF-2α protein in the portal area, and it is difficult to identify the hepatic sinus because of swelling of hepatocytes. It seems that the two studies have avoided reporting the expression of HIF-2α protein in the whole hepatic lobule, especially in portal area. In our study, we constructed a model of hepatocyte-specific HIF-2α knockout mice. We reported that the hepatocyte-specific knockout of HIF-2α could not alleviate CCl_4_-induced liver fibrosis because the elevated HIF-2α expression was mainly concentrated in hepatic sinuses and portal areas, but not in hepatocytes. Therefore, CCl_4_-induced liver fibrosis has a greater impact on the expression of HIF-2α in endothelial cells than in hepatocytes. In fact, according to the evidence provided by Wang et al. (2017), increases in HIF-2α protein levels are mainly localized to hepatic sinus endothelial cell close to the portal area in mice with CCl_4_-induced liver fibrosis, which is consistent with our results.

Interestingly, hepatocyte-specific HIF-2α deficiency did not affect serum lipid levels in mice without any treatments, but the serum TC and TG levels were improved by HIF-2α deficiency in mice injected intraperitoneally with olive oil. Additionally, hepatocyte-specific HIF-2α deficiency protected the hepatic lipid synthesis function and led to a higher TC and TG levels in mice with liver fibrosis. Therefore, hepatocyte-specific HIF-2α deletion regulates the lipid metabolism in hepatocytes and maintains the stability of serum lipids level, as suggested by the results from previous research by our team (Chen et al., 2019). Increases in fatty acid metabolism caused by NAFLD in hepatocytes consumes a large amount of oxygen. It is speculated that hypoxia may be mainly concentrated in hepatocytes, but has little effect on the oxygen supply of hepatic sinus. However, the liver fibrosis induced by CCl_4_ may be different. The toxicity of CCl_4_ first spreads to the hepatic sinuses through the portal area, and then enters the terminal hepatocytes. In addition, the transcriptional level of HIF-2α in endothelial cells is higher than that in hepatocytes. Therefore, the hypoxia of endothelial cells in the hepatic sinuses and portal area may be more severe, or the endothelial cells may be more sensitive to hypoxia, than that of hepatocytes. Therefore, the differences in the liver fibrosis models induced by CCl_4_ and NAFLD may also lead to different results.

There are some limitations in this study. First, hepatocyte-specific knockout of HIF-2α may not affect the continued action of HIF-1α, and we did not evaluate the expression of HIF-1α in a fibrotic liver. However, it will not change the conclusion that hepatocyte-specific deletion of HIF-2α cannot alleviate liver fibrosis. Second, to imitate a person in a sick state as much as possible, the mice were not treated with hypoxia. In CCl_4_-stimulated mice, we observed a robust increase in the protein expression of HIF-2α in the liver, which indicates that the disease itself can lead to hypoxia in liver cells. Therefore, further research is needed in the future.

## Conclusions

HIF-2α is located mainly in sinusoidal endothelial cells instead of hepatocytes. Knockout of HIF-2α in hepatocytes does not alleviate CCl_4_-induced liver injury and fibrosis in mice. Therefore, hepatocyte HIF-2α expression may not be a key factor in the initiation of liver fibrogenesis, and hepatocyte-specific deletion of HIF-2α may not be the ideal therapeutic strategy for liver fibrosis.

## Supplemental Information

10.7717/peerj.15191/supp-1 Supplemental Information 1 Summary information regarding the datasets from the GEO database. Abbreviations: HBV, hepatitis B virus; HCV, hepatitis C virus; NAFLD, nonalcoholic fatty liver disease; CCl_4_, carbon tetrachloride; scRNA, single-cell RNA sequencing.

10.7717/peerj.15191/supp-2 Supplemental Information 2 Serum and liver tissue indicators.

10.7717/peerj.15191/supp-3 Supplemental Information 3 Mouse growth indicators.

10.7717/peerj.15191/supp-4 Supplemental Information 4 Author checklist.

## Additional Information and Declarations

### Data Availability

The following information was supplied regarding data availability: The raw measurements are available in the Supplemental Files.
