# Supplementary material for: Hepatocyte-specific knockout of HIF-2α cannot alleviate carbon tetrachloride-induced liver fibrosis in mice
Source: PeerJ. 2023 Apr 3;11:e15191. doi: 10.7717/peerj.15191 (PMC10078453; doi:10.7717/peerj.15191)
Supplement: Supplemental Information 1 — Abbreviations: HBV, hepatitis B virus; HCV, hepatitis C virus; NAFLD, nonalcoholic fatty liver disease; CCl4, carbon tetrachloride; scRNA, single-cell RNA sequencing. [file peerj-11-15191-s001.docx]

| Table S1: Summary information regarding the datasets from the GEO database | | | | | | | |
| --- | --- | --- | --- | --- | --- | --- | --- |
|  | Data type | Organism | Hepatopathy | Experimental group | | Control group | |
|  |  |  |  | Cases | N | Cases | N |
| GSE142530 | RNA-seq | Human | Alcoholic | Cirrhosis | 6 | Hepatitis | 10 |
| GSE84044 | Array | Human | HBV | Advanced fibrosis | 61 | Mild fibrosis | 63 |
| GSE33650 | Array | Human | HCV | Fibrosis | 24 | No fibrosis | 30 |
| GSE49541 | Array | Human | NAFLD | Advanced fibrosis | 32 | Mild fibrosis | 40 |
| GSE207855 | RNA-seq | Mouse | CCl_4_ | Intraperitoneal injection of CCl_4_ three times per week for 6 weeks | 6 | Intraperitoneal injection of oil three times per week for 6 weeks | 6 |
| GSE134037 | scRNA | Mouse | CCl_4_ | Intraperitoneal injection of CCl_4_ two times per week for 6 weeks (liver nonparenchymal cells) | 1 | Intraperitoneal injection of oil two times per week for 6 weeks (liver nonparenchymal cells) | 1 |
| Abbreviations: HBV, hepatitis B virus; HCV, hepatitis C virus; NAFLD, nonalcoholic fatty liver disease; CCl_4_, carbon tetrachloride; scRNA, single-cell RNA sequencing. | | | | | | | |
